# Maternal Risk Factors for Oral Clefts: A Case-Control Study

**Published:** 2012

**Authors:** Mohammad Jafar Golalipour, Nafiseh Kaviany, Mostafa Qorbani, Elham Mobasheri

**Affiliations:** 1 *Gorgan congenital malformations research center, Golestan university of Medical sciences, Gorgan, Iran.*; 2 *Department of Epidemiology, Tehran University of Medical Sciences, Tehran, Iran.*; 3 *Department of Gynecology, Gorgan Congenital Malformations Research Center, Golestan University of Medical, Sciences, Gorgan, Iran.*

**Keywords:** Cleft lip, Cleft palate, Consanguinity, Ethnicity, Folic acid, Parity

## Abstract

**Introduction:**

A cleft lip with or without a cleft palate is one of the major congenital anomalies observed in newborns. This study explored the risk factors for oral clefts in Gorgan, Northern Iran.

**Materials and Methods:**

This hospital-based case-control study was performed in three hospitals in Gorgan, Northern Iran between April 2006 and December 2009. The case group contained 33 newborns with oral clefts and the control group contained 63 healthy newborns. Clinical and demographic factors, including date of birth, gender of the newborns, type of oral cleft, consanguinity of the parents, parental ethnicity, and the mother's parity, age, education and intake of folic acid were recorded for analysis.

**Results:**

A significant association was found between parity higher than 2 and the risk of an oral cleft (OR= 3.33, CI 95% [1.20, 9.19], P> 0.02). According to ethnicity, the odds ratio for oral clefts was 0.87 in Turkmens compared with Sistani people (CI 95% [0.25, 2.96]) and 1.11 in native Fars people compared with Sistani people (CI 95% [0.38, 3.20]). A lack of folic acid consumption was associated with an increased risk of oral clefts but this was not statistically significant (OR = 1.42, CI 95% [0.58, 3.49]). There were no significant associations between sex (OR boy/girl = 0.96, CI 95% [0.41, 2.23]), parent familial relations (OR = 1.07, CI 95% [0.43, 2.63]), mother's age and oral clefts.

**Conclusions:**

The results of this study indicate that higher parity is significantly associated with an increased risk of an oral cleft, while Fars ethnicity and a low intake of folic acid increased the incidence of oral clefts but not significantly.

## Introduction

A cleft lip with or without a cleft palate is the most common orofacial congenital anomaly among live births (1). The rate of oral clefts varies from 1 in 500 births to 1 in 2,000 births in different countries (2). The rate of oral clefts in the north of Iran was reported to be 0.97 cases per 1000 live births between 1998 and 2003 (3). Oral clefts have a multifactorial origin and are affected by genetic and environmental factors. Gender, geographic location, nationality, nutritional and periconceptional consumption of folic acid have an effect on the incidence rate of oral clefts (4-13). It has also been reported that tobacco use, antiepileptic drugs and possibly alcohol consumption (14), low birth weight (15) and mustard gas (16) increase the incidence rate of oral clefts in newborns. Several studies have also reported that racial/ethnic factors (17,18) and consanguinity (15,19) have an effect on the incidence rate of oral clefts.

As the incidence rate of oral clefts is relatively high in Northern Iran (3), the population is racially/ethnically heterogeneous, and there have been no case-control studies regarding oral clefts in the region, this study was conducted to identify the risk factors for congenital cleft palate and cleft lip and palate in the north of Iran.

## Materials and Methods

This hospital-based case-control study was performed in three hospitals (Dezyani, Falsafi, Masoud) in Gorgan, which is located in the north of Iran. Ethical approval for the study was obtained from the ethics committee of Golestan University of Medical Sciences. Gorgan is the capital city of Golestan Province and the three hospitals in this study have an annual rate of more than 10,000 deliveries, which accounts for approximately 30% of deliveries in Golestan Province, Northern Iran. Golestan Province is on the south-eastern edge of the Caspian Sea. The region has a population of about 1.5 million people and covers an area of about 20,460 square kilometers. Fars, Turkman and Sistani people are the three main ethnic groups in Gorgan. Native Fars people are the predominant ethnic group in the area with the most members, the Turkman people are an ethnic group that emigrated from central Asia more than three centuries ago, and the Sistani group emigrated from southeastern Iran half a century ago.

Between April 2006 and December 2009, 33 newborns with oral clefts and 63 normal newborns (control group) and their mothers were evaluated. To form the control group, for every case we selected the next one or two healthy infants that were born.A consent form was completed by the parents of all the newborn infants in the study. 

All live births during the investigation were examined and screened for cleft lip and cleft palate immediately after delivery by a gynecologist. The diagnosis was later confirmed by a pediatrician according to ICD10. 

A questionnaire addressing the relevant clinical and demographic factors for each case and control subject was completed by the pediatrician and by a nurse during an interview with the parents. The questionnaire data included birth date and gender of the infant, type of oral cleft, consanguinity of the parents, parental ethnicity, and the mother's parity, age, education and intake of folic acid. Data were collected through interviews with mothers in the immediate postpartum period, as well as by consulting the patient records of both the mothers and newborn infants. 

The data analysis was performed using SPSS version 16. To investigate the factors affecting the occurrence of a cleft lip and/or palate, a logistic regression model was used to measure the crude odds ratio (OR) of the occurrence of a cleft lip and/or palate for each of the independent variables. The results are expressed as an OR with 95% confidence interval (CI). The significance level was set as P<0.05.

## Results

Out of 30,308 deliveries in the three hospitals in Gorgan between April 2006 and December 2009, 33 newborns with oral clefts were included in the study as cases and 63 healthy newborns as controls. The association of the evaluated risk factors with the occurrence of a cleft lip and/or palate is depicted in (Table 1). 

**Table 1:** Association between sociodemographic characteristics and congenital cleft lip and/or palate malformation in the case group (n= 33) and control group (n= 63) of a case-control study in Gorgan, Northern Iran

**Risk Factor**	**Case group** **N (%)**	**Control group** **N (%)**	**OR**	**CI 95%**	**P**
Parity					
1	11 (33.3)	34 (54)	1	-	-
2	8 (24.2)	16 (25.4)	1.54	0.521, 4.58	0.433
>2	14 (42.4)	13 (20.6)	3.329	1.2, 9.19	0.02
					
Familial marriage					
No	22 (66.7)	43 (68.3)	1	-	-
Yes	11 (33.3)	20 (31.7)	1.07	0.438, 2.63	0.874
					
Maternal age					
<20	5 (15.2)	5 (7.9)	1	-	-
20–34	24 (72.7)	54 (85.7)	0.44	0.118, 1.68	0.232
≥35	4 (12.1)	4 (6.3)	1	0.156, 6.42	1
					
Took folic acid					
Yes	9 (27.3)	23 (36.5)	1	-	-
No	24 (72.7)	40 (63.5)	1.42	0.583, 3.49	0.435
					
Residency					
Rural	18 (54.5)	33 (52.4)	1	-	-
Urban	15 (45.5)	30 (47.6)	0.917	0.394, 2.13	0.84
					
Gender (infant)					
Girl	15 (45.5)	28 (44.4)	1	-	-
Boy	18 (54.5)	35 (55.6)	0.96	0.412, 2.23	0.925
					
Maternal ethnicity					
Sistani	11 (33.3)	20 (31.7)	1	-	-
Turkmen	7 (21.2)	16 (25.4)	0.875	0.25, 2.98	0.831
Fars	15 (45.5)	27 (42.9)	1.11	0.386, 3.2	0.845
					
Maternal education					
Low level of education	1 (3)	10 (15.9)	1	-	-
Less than a diploma	24 (72.7)	33 (52.4)	7.27	0.871, 60.7	0.067
Diploma	6 (18.2)	18 (28.6)	3.33	0.35, 31.74	0.295
More than a diploma	2 (6.1)	2 (3.2)	10	0.584, 171.2	0.112

The mean maternal age in the case and control group was 25.9 and 25.1 years, respectively. There was no association between maternal age and increased risk for oral clefts. Oral clefts were found to be more common in males than females, but there was no significant association between infant gender and oral clefts. 

A total of 33.3% of infants with a cleft lip and/or palate were the result of consanguineous marriages, whereas this rate was 31.7% in the control group. There was no association between consanguineous marriages and an increased risk of oral clefts (OR = 1.07, CI 95% [0.438, 2.63], P < 0.87). However, in mothers with parity greater than 2 there was a significantly increased risk of having a child with an oral cleft compared with mothers whose parity was 1 (OR = 3.33, CI 95% [1.2, 9.19], P < 0.02). In terms of ethnicity, the odds ratio for oral clefts in infants of Turkmen ethnicity was 0.87 (CI 95% [0.25, 2.96]) and 1.11 in infants of native Fars ethnicity (CI 95% [0.39, 3.2]) compared with infants of Sistani ethnicity. A total of 63.5% of mothers in the control group and 72.7 % in the case group did not take folic acid before conceiving or during pregnancy. A lack of folic acid consumption was associated with an increased risk of oral clefts (OR= 1.42, CI 95% [0.58, 3.49]) but this was not significant. A total of 54.5% of mothers with affected newborns lived in a rural area and 45.5% in an urban area but this was not associated with an increased risk of oral clefts (OR urban/rural = 0.917, CI 95% [0.39, 2.13]).

## Discussion

In this study, oral clefts are more common in males but this result was not statistically significant indicating there is no associated between gender and oral clefts. This result is similar to other studies in Japan (20) and Tehran, Iran (21). Ethnicity was also not a risk factor for oral clefts in our study but other studies have reported that ethnicity plays a significant role in the prevalence of oral clefts (17, 22). The limited sample size in our study may be a cause of this difference.

According to our results, no association was found between maternal age and oral clefts. This result is similar to studies by Jagomagi and colleagues (23) and Fathololumi and colleagues (24), as well as a study by Abramowic and colleagues, which reported that there was no association between the type of cleft and maternal age (P > 0.07) (25). On the other hand, Vallino-Napoli and colleagues (26), Elahi and colleagues (27) and Bille and colleagues (28) reported that the incidence of cleft lip and/or palate probably increases with maternal age. The study by Vallino-Napoli and colleagues in Australia between 1983 and 2000 reported that when cleft lip with and without cleft palate was considered as one group, the data showed a significantly increased adjusted odds ratio (OR=51.63, 95% CI [51.1, 2.5]) for women who were 40 years old, compared with all other ages (26). A study by Bille and colleagues in Denmark also showed that both high maternal and paternal age were associated with cleft lip with or without cleft palate and higher paternal age but not maternal age increased the risk of cleft palate only (28).

There was also no association between consanguinity and oral clefts but several studies in Pakistan (27), Tehran, Iran (21,29), and South India (30) have reported a significant association between familial matrimony and orofacial clefts. A study by Azimi and Karimian in Tehran showed that consanguineous marriage seems to have a significant role (P=0.02) in the prevalence of oral clefts (29). One risk factor that did show an association with an increased risk of oral clefts was the parity of the mother. Parity higher than 2 was significantly associated with an increased risk of oral clefts. Our result is not agreement with the study by Abramowicz and colleagues (25), which reported that there was no significant association between the type of cleft and maternal parity (P>0.07) (30). The association we observed between parity and an increased risk of oral clefts may be due to the physiological condition of the mother, as increased parity may have adverse effects on micronutrients and subsequently increase the risk of oral clefs.

In our study folic acid consumption was not significantly associated with oral clefts. However, Van Rooij and colleagues in the Netherlands (11) reported a significant reduction in the risk of cleft lip and/or palate with the use of folic acid supplements. 

Also, Wilcox and colleagues reported a 39% decrease in the risk of cleft lip and palate with the use of folic acid supplements adjusted for the use of multivitamins (31). 

Based on our findings, there is also no significant association between maternal education and the risk of oral clefts or between residency in a rural versus urban area and the risk of oral clefts. Our finding regarding maternal education is similar to a study by Lebby and colleagues (32) and is in contrast with a study in India by Reddy and colleagues (30). Lebby and colleagues reported that maternal education did not achieve the requisite level of significance in population samples (32), but Reddy and colleagues showed that maternal education is related to oral clefts. (30). Our finding regarding residency type is similar to a study in China (33) and is in contrast with a study in India (30).

The limitation of this study was the sample size. We also need to maintain an accurate database for cleft registrations; systematic record keeping is essential in this area. 

## Conclusions

The present study showed that ethnicity and a lack of folic acid intake was not significantly associated with an increased risk of oral clefts in infants, however high parity was significantly associated with an increase in the rate of oral clefts. Further studies with a larger sample size including all the hospitals in Golestan Province in Northern Iran are required.
